# Antivirus effectiveness of ivermectin on dengue virus type 2 in *Aedes albopictus*

**DOI:** 10.1371/journal.pntd.0006934

**Published:** 2018-11-19

**Authors:** Tie-Long Xu, Yin Han, Wei Liu, Xing-Ya Pang, Bin Zheng, Yi Zhang, Xiao-Nong Zhou

**Affiliations:** 1 National Institute of Parasitic Diseases, Chinese Center for Disease Control and Prevention, and National Center for Tropical Diseases Research, Shanghai, the People’s Republic of China; 2 Key Laboratory of Parasite and Vector Biology, Ministry of Public Health, and WHO Collaborating Centre for Tropical Diseases, Shanghai, the People’s Republic of China; 3 Nanchang Customs District, and Jiangxi International Travel Healthcare Center, Nanchang, the People’s Republic of China; Saudi Ministry of Health, SAUDI ARABIA

## Abstract

**Background:**

Dengue fever is the most rapidly spreading mosquito-borne viral disease over the past 50 years, with a 30-fold increase in global incidence. Dengue vector control is a key component for the dengue control strategy, since no absolutely effective vaccine or drug is available yet. However, the rapid rise and spread of mosquito insecticide resistance have become major threats to the efficiency of insecticide-based vector control activities. Thus, innovative vector control tools are badly needed. This study aims to confirm the antivirus effectiveness of ivermectin on dengue virus type 2 (DENV-2) in *Aedes albopictus* (Skuse, 1894), then to explore its potential use in the combating to the dengue epidemics.

**Methods:**

*Aedes albopictus* were first infected with DENV-2 in human whole blood, and at the fourth day after infectious blood feeding, they were divided into eight groups. Seven of them were held for six days with access to 0, 2, 4, 8, 16, 32 and 64 ng/ml ivermectin, respectively, and the last one was set as a historical control group, which was stored at -80°C until being detected at the same time with the other groups. Each mosquito was detected using real-time fluorescent RT-PCR kit. DENV-2 RNA concentration (copies/ml) and infection rate in each group were compared.

**Results:**

Both of quantitatively and qualitatively inhibiting effects of ivermectin have been detected in this study. Generally, DENV-2 replicated well in *Aedes albopictus* without ivermectin intervention, whose virus loads exhibited significantly higher when the mosquitoes were holding from 4 days to 10 days after infectious blood feeding. In contrast, with the treatment of ivermectin, the infection rate was reduced by as much as 49.63%. The regression equation between infection rates (Y_2_) and ivermectin concentration log_2_ values (X_2_) was obtained as Y_2_ = 91.41–7.21*X_2_ with R^2^ = 0.89.

**Conclusion:**

Ivermectin can directly or indirectly inhibit DENV-2 multiplication in *Aedes albopictus*. Moreover, the actual concentration for application in zooprophylaxis needs to be confirmed in the further field trials.

## Author summary

Dengue fever is one of neglected vector-borne tropical diseases with a 30-fold increase in global incidence recently. In 2012, World Health Organization set a goal to reduce dengue mortality by at least 50% by 2020. Being faced with more challenges in the dengue control programs, such as the increase of dengue outbreaks, lacking absolutely effective vaccine, rise of vector insecticide resistance and so on; innovative vector control tools are urgently needed for current control programs on dengue fever. To find a new avenue in vector control, we for the first time assessed the inhibiting effectiveness of ivermectin on dengue virus type 2 (DENV-2) inside *Aedes* mosquitoes. We found that about 80% *Aedes albopictus* mosquitoes were effectively infected with DENV-2 without treatment of ivermectin. But in the groups of ivermectin treatment, the infection rate of DENV-2 and the median of virus loads were significantly reduced by up to 49.63% and 99.99%, respectively. Both quantitatively and qualitatively inhibiting effects of ivermectin were detected. We found out that ivermectin was able to effectively inhibit the DENV-2 multiplication in *Aedes albopictus*, which may gave us a hint that using ivermectin in some control programs as a zooprophylaxis to block dengue epidemic through inhibiting DENV-2 in field *Aedes* mosquitoes.

## Introduction

Dengue fever is the most rapidly spreading mosquito-borne viral disease over the past 50 years, with a 30-fold increase in global incidence [1]. To reverse the growing trend, comprehensive technical strategies involving diagnosis and case management, integrated surveillance and outbreak preparedness, sustainable vector control and future vaccine implementation are necessary. Apart from the other technical elements, effective vector control is a critical component to achieve and sustain reduction of morbidity attribute to dengue. There are well-documented and various historical examples of dengue elimination or significant reduction through control of *Aedes aegypti* (Linnaeus and Hasselquist, 1762) [2]. While bioassay demonstrates that resistance to organophosphates and pyrethroids are widespread in *Aedes aegypti* and *Aedes albopictus* [3–9]. Therefore, innovative vector control tools are badly needed for current control programs on dengue fever [1, 10]. Many new tools in vector control have been developed, such as insecticide-treated materials [11–14], lethal ovitraps [15, 16], spatial repellents [12, 14], genetically modified mosquitoes [17–19], *Wolbachia*-infected *Aedes* spp. [20], and so on. But effective tools able to block the transmission of dengue inside vector are still lacking. Therefore, we are trying to find an innovative avenue to inhibit dengue virus development inside *Aedes* mosquito in order to block the cycle of dengue transmission.

Two significant progresses in the tools to block the transmission of dengue inside vector benefit from the advances in genetic engineering technology and molecular biology. One is the discovery of cytoplasmic incompatibility (CI) induced by the intracellular bacteria *Wolbachia* (Hertig and Burt, 1924), which has enhanced replacement in the control programs [21, 22]. CI is a reproductive phenotype induced by bacterial endosymbionts in arthropods. Measured as a reduction in egg hatchability resulting from the crossing of uninfected females with bacteria-infected males, CI increases the frequency of bacteria-infected hosts by restricting the fertilization opportunities of uninfected hosts in populations [23]. Markedly reduced severity of dengue virus infection has been found in *Aedes albopictus* infected with *Wolbachia* [21, 24]. The other one is the introduction of genetic-based strategies, which has the goal to eliminate or reduce mosquito densities below transmission threshold through population suppression or to establish mosquito populations that are refractory to the pathogen through population replacement and/or modification [25]. Genetically modified *Aedes aegypti* mosquitoes that activate the conserved antiviral JAK/STAT pathway in the fat body tissue have been developed, and the modified population inhibits infection with several dengue virus (DENV) serotypes [26], but its use encounters regulatory barriers and public opposition in some countries. Few drugs have been tested to inhibit the virus transmission inside mosquito, although some drugs against dengue virus effectively in vitro have been reported, such as quercetin [27], ivermectin [28–30], dasatinib [31], pyran naphthoquinones [32], mycophenolic acid [33, 34], castanospermine [34], deoxynojirimycin [35, 36]. From these drugs, we choose ivermectin as an available compound for the investigation by considering following three facts: (i) ivermectin has been used for about 30 years for treatment of parasitic infections in human since 1988 [37], and ivermectin mass drug administration (MDA) to humans has been suggested as a possible vector control method to reduce *Plasmodium* transmission [38–40]; (ii) ivermectin has the ability to target exophagic and exophilic vectors [40, 41] with a different mode of action [42, 43] from the currently used insecticides [44], and then avoid known mosquito behavioral and physiological resistance mechanisms [45]; (iii) ivermectin is an inhibitor for the development of dengue virus in cells [28–30]. The purpose of this investigation is to further determine ivermectin efficacy against dengue virus type-2 (DENV-2) in *Aedes albopictus*, and explore its potential application as an innovative vector control tool.

## Materials and methods

### Ethics statement

The study was approved by the ethical review committee of National Institute of Parasitic Diseases, Chinese Center for Disease Control and Prevention, and approval document number was 20160627. Moreover, no specific permits were required for the described field studies. The studies did not involve endangered or protected species.

### Virus and cells

C6/36 mosquito cell and BHK-21 cell lines, derived from *Aedes albopictus* and Baby Hamster Syrian Kidney respectively, were used in this study. The cell lines were maintained and propagated in Dulbecco’s Modified Eagle Medium (DMEM) (Gibco by Life technologies, Australia) containing 10% (v/v) fetal bovine serum (FBS) (Gibco by Life technologies, Australia) and 1% (v/v) Penicillin-Streptomycin (Gibco by Life technologies, Australia). Cultured C6/36 was incubated at 28°C in 5% CO_2_ humidified chamber, and was passaged every 2~3 days. At the time of virus multiplication, the serum concentration was reduced to 2% and temperature was increased to 33°C. DENV-2 was propagated using C6/36 cell line and harvested after CPE presentation on day five post-infection. Supernatants containing DENV-2 were collected, centrifuged at 4,000 xg for 10 minutes to clear cellular debris, and then were stored at -80°C until further use. The titer of viral stocks was measured by TCID (50) % using serial dilutions of 10^1^ to 10^6^ of the viral stocks inoculated into BHK-21 cells. The viral titer was calculated according with Reed and Munch [46]. Cell lines and virus were kindly provided by Shenzhen Center for Disease Control and Prevention (Shenzhen, China).

### Mosquito

Adult mosquitoes of *Aedes albopictus* were obtained from the National Institute of Parasitic Diseases at Chinese Center for Disease Control and Prevention based in Shanghai and were raised at 26±2°C, 60~80% relative humidity, and a 12:12 light: dark cycle. The larvae were raised on a diet of rat food. Adults were provided with 10% (g/v) sucrose solution. Adult mosquitoes aged between three and five days post emergence from larvae were used as experiment objects.

### Experiment of *Aedes* mosquito fed with ivermectin

The powdered ivermectin formulation was obtained from Sigma-Aldrich (St. Louis, MO). Ivermectin was diluted in dimethyl sulfoxide (DMSO) to 10 mg/ml and aliquots were frozen at −20°C. Frozen aliquots of ivermectin were thawed and serially diluted in phosphate buffered saline (PBS) prior to addition to human whole blood heated to 37°C prior to mixing. 10 μl of varied concentrations of ivermectin in PBS were added to 990 μl of human whole blood meal to reach 0, 2, 4, 8, 16, 32 and 64 ng/ml concentrations offered to mosquitoes.

*Aedes albopictus* aged between three and five days post emergence from larvae were fed together with human whole blood containing the same titer of DENV-2. After blood feeding, all fully engorged mosquitoes were gently transferred by aspiration to a new 3L cardboard cartons and held in an incubator at 26±2°C, 60~80% relative humidity, and a 12:12 light: dark cycle. Engorged mosquitoes were held for four days with access to human whole blood, and then were randomly divided into eight groups. Seven of them were held for six days with access to 0, 2, 4, 8, 16, 32 and 64 ng/ml ivermectin, respectively, and the last one was set as a historical control group. The mosquitoes in the historical control group were stored at -80°C until being detected at the same time with mosquitoes in the other groups. In this way, one parallel control group (0ng/ml), one historical control group and six treatment groups were set. Three replicates were performed for each group/concentration, with at least 20 mosquitoes per replicate being analyzed. The human whole blood was obtained from Jiangxi International Travel Healthcare Center, which provided healthy physical examination for community.

### Examination of ivermectin efficacy on DENV-2 in *Aedes albopictus*

Mosquitoes treated as described above were collected, and DENV-2 RNA copies in each mosquito were detected by real-time RT-PCR at the same time, and the cycle threshold (C_T_) value of each mosquito was recorded. At least 60 mosquitoes were analyzed for each group.

After being frozen to death at −20°C, each mosquito was collected in a grinding tube with 350 μl lysis buffer and then was fully grinded by tissue grinded instrument. The DENV-2 RNA was isolated with RNeasy plus Mini Kit (250) (Qiagen, German), and quantitatively tested with the dengue virus 2 real-time fluorescent RT-PCR kit (Shanghai ZJ Bio-Tech, China). The Master Mix volume for each reaction was pipetted as follows: super mix 18 μl, enzyme mix 1 μl, internal control 1 μl, extraction RNA 5 μl. PCR reaction conditions were: one cycle of 45°C for 10 minutes and 95°C for 15 minutes, then 40 cycles of 95°C for 15 seconds and 60°C for 60 seconds, fluorescence measured at 60°C. During the bioassay, the standard curve between C_T_ values and DENV-2 RNA concentrations (copies/ml) was also detected as described previously [47].

### Statistical analysis

The standard curve between C_T_ values and DENV-2 RNA concentrations (copies/ml) was analyzed by linear correlation regression with regression equation and the DENV-2 RNA concentration (copies/ml) in each mosquito were calculated by the C_T_ value according to the regression equation. All the DENV-2 RNA concentration (copies/ml) in each group were presented by the key parameters, including the median, 75^th^ percentile (P_75_), 25^th^ percentile (P_25_), maximum (Max), minimum (Min) and inter-quartile range (Q). For the DENV-2 RNA copies, the differences among the eight groups were analyzed by Kruskal-Wallis test (K-W test), and then were further analyzed by the Turkey studentized range test to determine exactly which two groups had significant difference. According to the detection reagent protocol, when the C_T_ value of mosquito was less than or equal to 40.00, the mosquito was judged to be positive with DENV-2, and the infection rate in each group was calculated. For the infection rates, Chi-squared test (*χ*^2^ test) was used to examine the statistical significances among the eight groups, and Duncan multiple range tests were used to determine pair-wise differences, and then linear correlation regression method was used to further analyze the correlation between the infection rates and ivermectin concentrations. P < 0.05 was considered to be significant.

## Results

### Relationship between C_T_ values and DENV-2 RNA concentrations

The DENV-2 RNA concentration of positive control sample from the commercial kit was 10,000,000 copies/ml, which was serially diluted to 1,000,000, 100,000, 10,000, 1,000, 100 copies/ml. They were synchronously detected with mosquito samples. Three replicates were performed for each concentration. The relationship between C_T_ values (X_1_) and log_10_ values of DENV-2 RNA concentrations (Y_1_) was expressed by the regression equation, which was obtained from the experimental data as Y_1_ = 12.70–0.28*X_1_ with R^2^ = 0.99. Thus, we got the concentration of DENV-2 RNA in each mosquito by the standard curve.

### Development of DENV-2 inside *Aedes albopictus* without ivermectin treatment

The infection rate in the mosquitoes fed with 0 ng/ml ivermectin (parallel control group) was 84.62%, which was not significantly higher than the infection rate (81.67%) in the historical control group (Table 1). And the mosquitoes fed with 0 ng/ml ivermectin were of higher DENV-2 RNA concentrations than mosquitoes in historical control group (Table 2), verifying the multiplication of DENV-2 inside *Aedes albopictus* when they were raised from 4 days to 10 days post infectious blood feeding without ivermectin intervention.

**Table 1:** Pair-wise differences of infection rates among the eight groups.

Group*	Engorged mosquito (n)	Positive mosquito (n)	Infection rate (%)	Average of infection rate** (%)
Historical control	20	17	85.00	81.67^a^
	20	16	80.00	
	20	16	80.00	
0ng/ml	21	17	80.95	84.62^a^
	21	19	90.48	
	23	19	82.61	
2ng/ml	25	22	88.00	85.29^a^
	20	17	85.00	
	23	19	82.61	
4ng/ml	20	16	80.00	82.54^a^
	21	18	85.71	
	22	18	81.82	
8ng/ml	20	14	70.00	74.24^a, b^
	25	19	76.00	
	21	16	76.19	
16ng/ml	20	13	65.00	63.33^b, c^
	20	13	65.00	
	20	12	60.00	
32ng/ml	23	12	52.17	54.29^c, d^
	24	13	54.17	
	23	13	56.52	
64ng/ml	20	9	45.00	42.62^d^
	20	8	40.00	
	21	9	42.86	

*: 0, 2, 4, 8, 16, 32, 64ng/ml are the groups of mosquitoes fed with according concentrations of ivermectin.

**: The average of infection rate that does not share a same letter indicates statistical difference at P < 0.05 using the Duncan multiple range test.

**Table 2:** Pair-wise differences of DENV-2 RNA concentrations (copies/ml) among the eight groups.

Group*	Engorged mosquito (n)	Max	Min	Median	P_75_	P_25_	(Q)
historical control^a^	60	8.15x10^5^	21.52	2.23x10^4^	1.61x10^4^	80.5	1.59x10^4^
0ng/ml^b^	65	1.58x10^7^	21.52	2.01x10^6^	7.78x10^6^	135.01	7.78x10^6^
2ng/ml^b^	68	1.64x10^7^	21.52	1.94x10^6^	3.69x10^6^	78.81	3.69x10^6^
4ng/ml^b^	63	1.41x10^7^	21.52	3.41x10^5^	2.44x10^6^	107	2.44x10^6^
8ng/ml^b^	66	9.06x10^6^	21.52	8.46x10^5^	2.61x10^6^	21.52	2.61x10^6^
16ng/ml^a^	60	3.75x10^6^	21.52	3.41x10^4^	3.39x10^5^	21.52	3.39x10^5^
32ng/ml^a^	70	2.67x10^6^	21.52	37.72	2.76x10^5^	21.52	2.76x10^5^
64ng/ml^a^	61	2.23x10^6^	21.52	21.52	103.74	21.52	82.22

*: Groups that share different letter indicates statistical difference at P < 0.05 using Turkey studentized range test; 0, 2, 4, 8, 16, 32, 64ng/ml are the groups of mosquitoes fed with according concentrations of ivermectin.

### Efficacy of ivermectin on DENV-2 infection rate in *Aedes albopictus*

**Fig 1:**
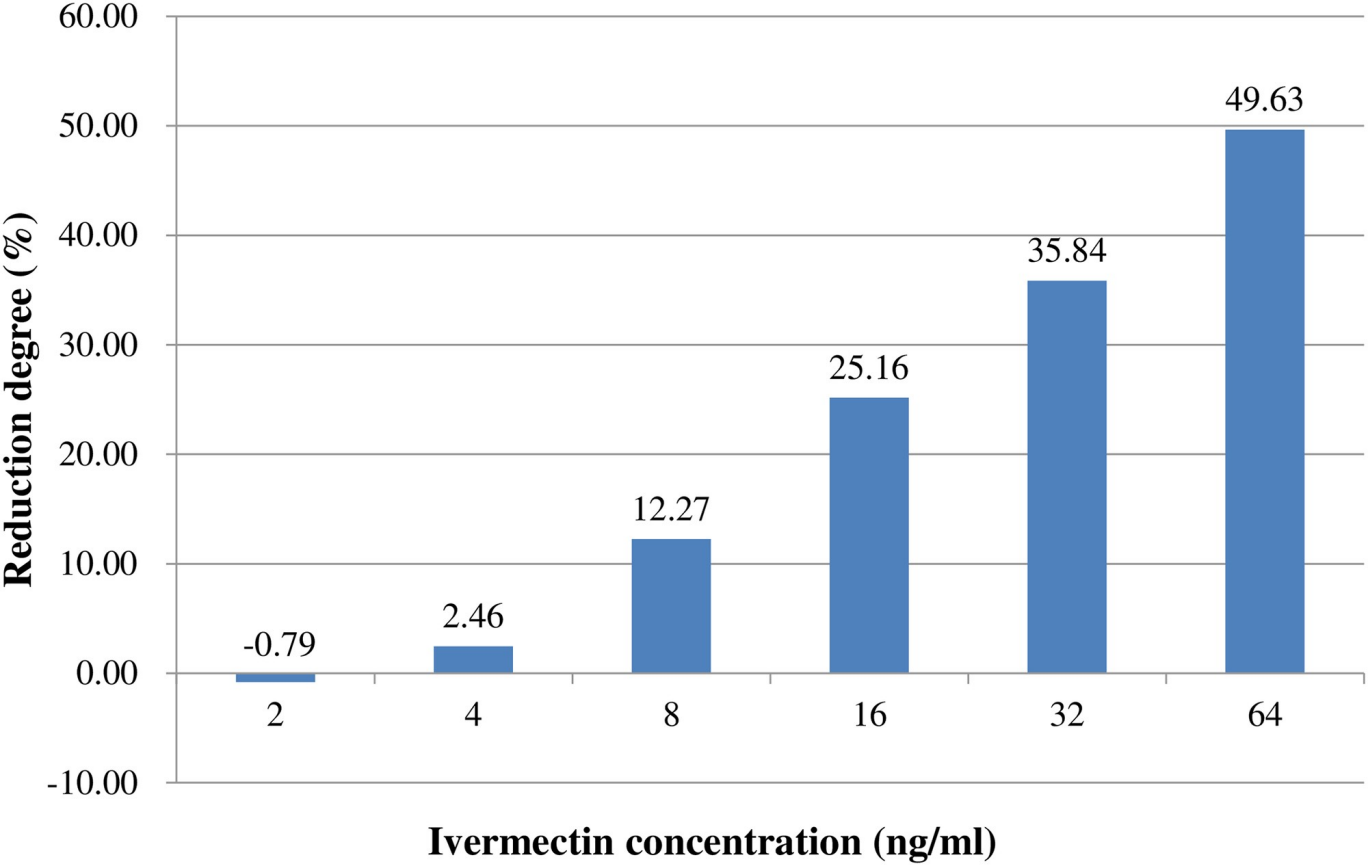
Compared to mosquitoes fed with 0ng/ml ivermectin, the reduction degree of infection rate for each treatment group. The calculation formula is as follows: (infection rate in mosquitoes fed with 0ng/ml ivermectin-infection rate in each treatment group)/ infection rate in mosquitoes fed with 0ng/ml ivermectin*100%.

**Fig 2:**
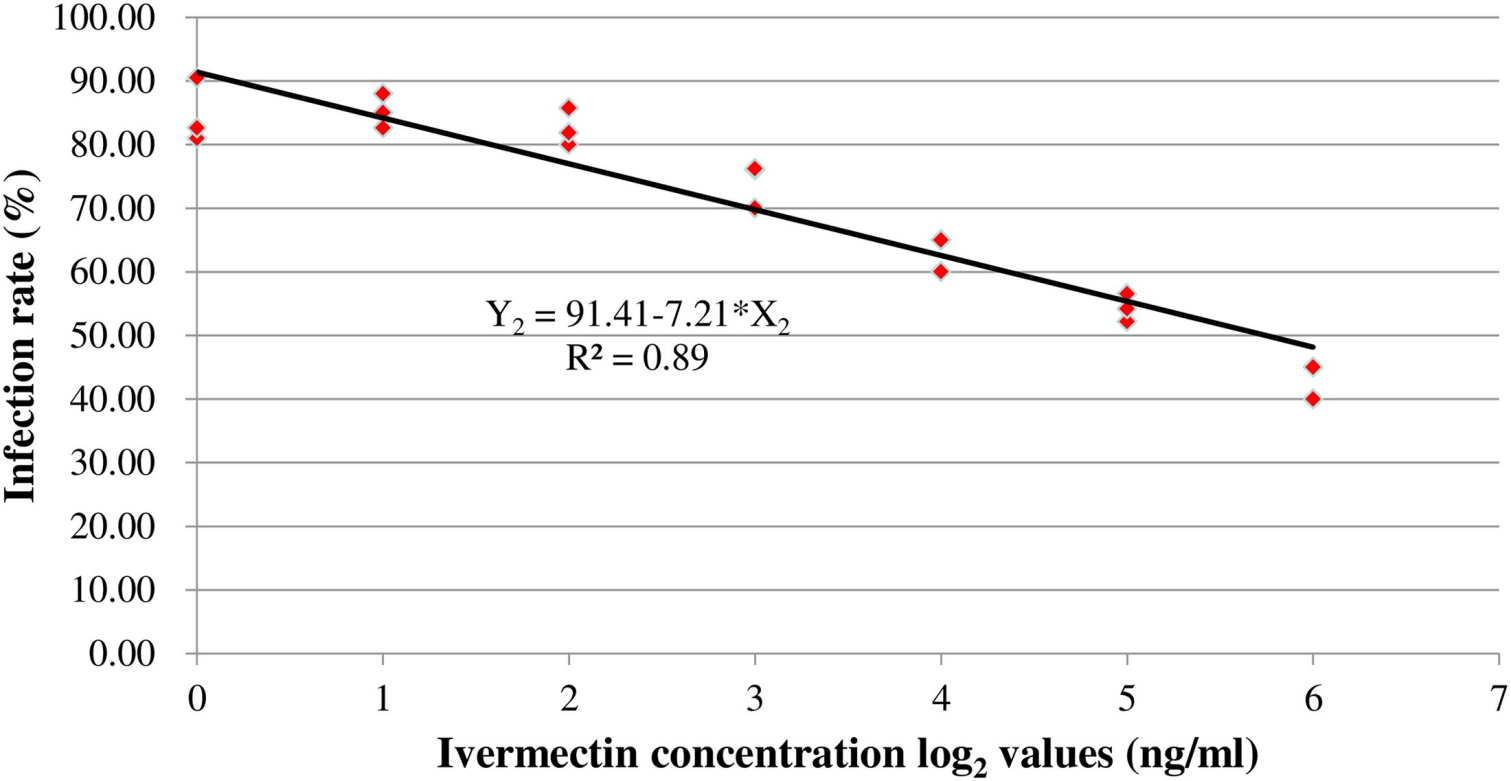
The correlation regression between infection rates and log_2_ values of ivermectin concentrations.

The average of infection rates in the seven groups treated with 0, 2, 4, 8, 16, 32 and 64 ng/ml ivermectin from 4 to 10 days post ingesting infectious blood were 84.62%, 85.29%, 82.54%, 74.24%, 63.33%, 54.29% and 42.62%, respectively, And the average of infection rates in historical control group was 81.67% (Table 1). Compared with the parallel control group or historical control group, infection rates in the mosquitoes fed with 2, 4, 8 ng/ml ivermectin were not significantly lowered; while infection rates in the mosquitoes fed with 16, 32, 64 ng/ml ivermectin were much lower (Table 1), with infection rate being reduced by as much as 49.63% (Fig 1). The regression equation between infection rates (Y_2_) and log_2_ values of ivermectin concentration (X_2_) was obtained as Y_2_ = 91.41–7.21*X_2_ with R^2^ = 0.89. (Table 1, Fig 2). What might confuse us here was that infection rate (85.29%) in mosquitoes fed with 2ng/ml ivermectin was seem to be higher than that in the historical control group (81.67%) or parallel control group (84.62%), but this differences were meaningless for being without statistical significance. In this part of experiment, antivirus effectiveness on DENV-2 in *Aedes albopictus* was observed in the ivermectin treatment groups at certain concentration, and the more ivermectin mosquito ingested, the lower the infection rate was.

### Efficacy of ivermectin on DENV-2 loads in *Aedes albopictus*

**Fig 3:**
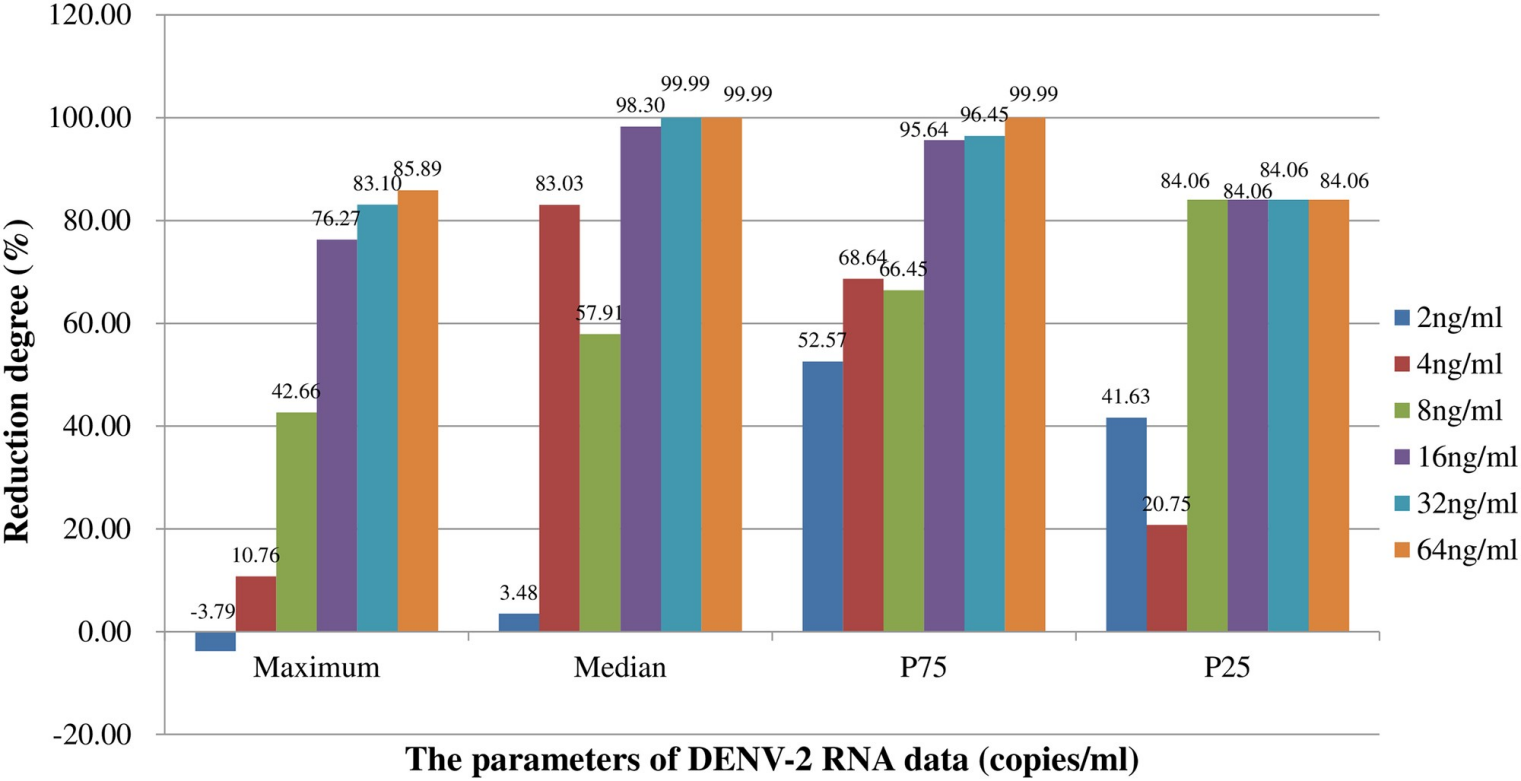
Compare to mosquitoes fed with 0ng/ml ivermectin, the reduction degree of maximum, median, P_75_, P_25_ of DENV-2 RNA concentration for each treatment group. The calculation formula is as follows: (parameter (e.g. median) in mosquitoes fed with 0ng/ml ivermectin-parameter in each treatment group)/ parameter in mosquitoes fed with 0ng/ml ivermectin*100%.

Related parameters indicating the DENV-2 loads in mosquitoes, including Max, median, P_75_, P_25_, Min and Q in each group were presented in Table 2. Compared with mosquitoes fed with 0 ng/ml ivermectin, mosquitoes fed with 2, 4, 8 ng/ml ivermectin carried the same level of DENV-2 RNA concentrations (copies/ml), and mosquitoes fed with 16, 32, 64 ng/ml ivermectin exhibited much lower DENV-2 RNA concentrations (copies/ml) (Table 2), with Max, median, P_75_ and P_25_ of DENV-2 RNA concentrations (copies/ml) being reduced by up to 85.89%, 99.99%, 99.99% and 84.06%, respectively (Fig 3). On the other hand, compared with mosquitoes in historical control group, DENV-2 had well developed inside mosquitoes fed with 0, 2, 4, or 8 ng/ml ivermectin showing significantly higher DENV-2 RNA concentrations (copies/ml), and was effectively inhibited in mosquitoes fed with 16, 32, or 64 ng/ml ivermectin showing the same level of DENV-2 RNA concentrations (copies/ml). The evidences confirmed the observation of antivirus effectiveness that virus loads in *Aedes albopictus* were statistically reduced by treatment of ivermectin when concentration of ivermectin was more than 16ng/ml. (Table 2)

## Discussion

In the past decades, dengue fever was a neglected vector-borne tropical disease, with few of control efforts to reduce the burden of the disease at national or international levels [1]. With more outbreaks occurred every year around the world [48–56], people are being faced with the problem of difficulty in blocking the growing trend of dengue transmission [1]. Currently, it has been a consensus that vector control is a key component in the dengue control programs. However, the rapid rise and spread of insecticide resistance have become major threats to the efficiency of insecticide-based vector control activities [1, 3–8]. It is an urgent need to develop innovative control tools for dengue vector control. It was our first try in the laboratory to find out whether ivermectin was able to effectively inhibit the DENV-2 multiplication in *Aedes albopictus* (Tables 1 and 2, Fig 2). The results give us a hint that using ivermectin in some strategy (e.g. zooprophylaxis [45]) is potentially a new way to stop dengue epidemic through inhibiting DENV-2 in field *Aedes* mosquitoes.

Interestingly, both of quantitatively and qualitatively inhibiting effects of ivermectin on DENV-2 have been detected in this study. Generally speaking, without ivermectin intervention, DENV-2 was well developed in *Aedes albopictus*, whose virus loads were significantly higher when the fully engorged mosquitoes were held from 4 to 10 days post infectious blood feeding (Table 2). In contrast, with the treatment of ivermectin, the infection rate and the median of DENV-2 RNA concentrations (copies/ml) were reduced by up to 49.63% and 99.99% (Figs 1 and 3). The linear correlation regression was established between concentration of ivermectin and infection rate of mosquitoes, and we found that 88.5% reduction of infection rate was attributed to the antivirus effectiveness of ivermectin (Fig 2). But the inhibiting effort of ivermectin on the virus in mosquitoes depended on the ivermectin dose, only when the ivermectin concentration was high enough (e.g. over 16ng/ml) can effectively inhibit DENV-2 inside *Aedes albopictus*. Thus, it is a new need to find out the exactly effective concentration of ivermectin per bite by mosquito as well as action mechanism of ivermectin in the future research, so as to guide its actual application in zooprophylaxis [45].

This study does not attempt to explore the action mechanism of ivermectin towards DENV-2 in *Aedes albopictus*. In our opinion, several potential reasons are leading to the inhibiting effect on any of the three aspects, namely virus, vector and natural microbiome of mosquitoes. Ivermectin is of a wide range of bioactivity [57]. It has been initially used in livestock or pets to kill parasites (e.g. gastrointestinal and mite) since 1981. Subsequently, it was proved to be very effective in humankind for a variety of internal nematode infections (e.g. Onchocerciasis) [37]. The action mechanism is that ivermectin targets glutamate-gated chloride channels, which plays fundamental roles in nematodes and insects while not accessible in vertebrates, leading to flaccid paralysis [37]. Ivermectin may also interact with γ-aminobutyric acid-gated chloride channels [58]. Both of the two channels are absent in virus. The antiviral activity of ivermectin towards dengue virus had been reported repeatedly since 2012 [29, 30], and then was confirmed in 2016 [28], but all of the researches were carried out in vitro. Considering the existed evidences, the antiviral mechanisms of ivermectin inhibiting DENV-2 in *Aedes albopictus* can be assumed from the following six aspects: (i) by targeting virus NS3 helicase activity [30]; (ii) by inhibiting nuclear import with respect to virus NS5 polymerase proteins [28]; (iii) by altering some aspects of the mosquito physiology, e.g. reducing the thickness of the peritrophic matrix in *Aedes aegypti* [59], delaying blood ingesting in *Anopheles gambiae* [60]; (iv) by stimulating enhanced anti-pathogen innate immunity, e.g. helping the host’s own immune response being able to overcome the immature worms and so kill them [61]; (v) by interacting with glutamate-gated chloride channels or γ-aminobutyric acid-gated chloride channels in the mosquito, and then reducing the adaptability between mosquito and pathogen; (vi) by influencing the natural microbiome of mosquitoes, since the natural microbiome, like *Wolbachia*, is related with the DENV 2 infection in *Aedes* mosquitoes [21, 22]. After all, these complex interactions between the pathogen and vector make it possible for ivermectin to have the function of antivirus inside *Aedes* mosquitoes. All of these potential reasons are worth more deep and overall follow-up study. Moreover, there are still some other effects remain poorly understood. It is unclear that how ivermectin exerts its effect on microfilariae infection in human [62] and *P*. *falciparum* in *Anopheles gambiae* (Giles, 1902) [63].

Ivermectin can block the DENV 2 at any anatomical barrier, like midgut or salivary gland. It was a great pity that we did not study where virus was blocked, so we did not test the viral infection, dissemination and transmission rates, all of which are always different in the same group of mosquitoes. Infection of mosquitoes requires the navigation of several anatomical barriers (e.g. the midgut and salivary glands barriers), and last is excreted into saliva for transmission to a new host. Escape from the midgut or colonization of the hemolymph does not necessarily guarantee the infection of the salivary glands. All of these barriers to productive infection of mosquitoes affect the transmission of viruses. Thus, transmission rate is always lower than viral infection rate. In this study, we just chose viral infection rates as an outcome measure. Maybe transmission rate is a more direct indicator to reflect the significance of ivermectin for the dengue control program in terms of blocking the dengue transmission. Anyway, the results showed that virus infection rates were significantly decreased by ivermectin (Table 1, Figs 1 and 2), which could also largely illustrate the above-mentioned significance of ivermectin. As shown in Table 1 and Fig 1, the virus infection rate in *Aedes albopictus* mosquitoes fed with 0ng/ml ivermectin was 84.62% (55/65), which was only 42.62% (26/61) in the mosquitoes fed with 64ng/ml ivermectin. The reduction degree of virus infection rate in the treated mosquitoes was up to 49.63%, which meant that there were more negative mosquitoes without virus disseminating from midgut to salivary gland, or that there were less positive mosquitoes with virus transmitting from mosquitoes to a new host. In this sense, we concluded that ivermectin can be used as alternative tool for controlling dengue vectors.

The results of our study may be quite meaningful for the dengue control program in terms of blocking the dengue transmission by using ivermectin. On one hand, the inhibiting effect on dengue virus in vivo means ivermectin which has been proved to be safety in human [64] has the potential to be developed as a drug for curing dengue patients. On the other hand, its antiviral effect inside the dengue vectors may lead to stopping the epidemics of dengue transmission in the field. Moreover, apart from the observed antivirus effect, ivermectin also is of insecticidal action [60, 65, 66]. For an example, about 32.22% (29/90) of mortality was observed in the mosquitoes fed with 64ng/ml ivermectin, which was much higher than 5.79% (4/69) of mortality in the mosquitoes fed with 0ng/ml ivermectin (*χ*^2^ = 16.58, df = 1, P<0.0001). Thus, it is an ideal drug for zooprophylaxis and endectocides [45]. Both of the strategies have been used in combating with malaria elimination [15, 16, 67], and resulted in a decrease of malaria incidence and prevalence in Pakistan [67]. The data illuminates that these two strategies may be still suitable for the dengue control program. Because of the antivirus and insecticidal effect, ivermectin using in endectocides can not only kill a number of blood-sucking vectors, but also inhibit the development of the dengue virus in the survived vectors, playing an unexpected role in reversing dengue’s growing trend in the world. However, the exact antivirus effectiveness and eventually being used in blocking dengue transmission need to be further validated with field *Aedes albopictus* mosquitoes or even other three serotypes dengue virus. Moreover, the actual concentration for application in zooprophylaxis needs to be confirmed in the field trials.

In conclusion, our study shows for the first time that ivermectin can directly or indirectly inhibit DENV-2 multiplication in *Aedes albopictus*. While the exact antivirus effectiveness and eventually being used in blocking dengue transmission need to be further validated in the field trials with field *Aedes albopictus* mosquitoes or even other three serotypes dengue virus.
