# Expression of Concern: Antivirus effectiveness of ivermectin on dengue virus type 2 in *Aedes albopictus*

**DOI:** 10.1371/journal.pntd.0010117

**Published:** 2022-01-11

**Authors:** The PLOS Neglected Tropical Diseases Editors

After this article [1] was published, a concern was raised that for each experimental treatment (group), the triplicate data reported in Table 1 align quite closely with the group average, with low deviation and no outliers. In response, the authors noted that that they had repeatedly performed preliminary experiments to optimize conditions that yielded consistent results. The authors provided PCR data (S1 File) and analysis results (S2 File) to support Table 1.

In reviewing this issue, *PLOS Neglected Tropical Diseases* obtained input from multiple members of our Editorial Board. All consulted editors questioned the feasibility of the Table 1 results. They advised that the table and supporting dataset report infection rates that are more consistent within and across cohorts for each dose than are expected; while the results are theoretically possible, the probability of obtaining the reported values is exceedingly low for this assay and biological system.

It was also raised that based on published studies [2–5], ivermectin may inhibit replication but is not expected to remove all viral RNA present in the system prior to treatment; this has implications for the ‘Positive mosquito (n)’ (and corresponding negative mosquito) data reported in Table 1. In response to this, the corresponding author commented that per [2–5] DENV-2 virus nucleic acid will be present for a long time after death, but this cannot be considered as the degradation rate of nucleic acid in live mosquitoes as used in [1] because in living organisms active enzymes metabolize viral nucleic acid [6–9].

Overall, the consulted editors advised that there are concerns about the feasibility, integrity, and reliability of the Table 1 results which were not resolved by the data and comments received in post-publication discussions. Therefore, the *PLOS Neglected Tropical Diseases* Editors issue this Expression of Concern.

In the post-publication assessment, it was also raised that detection of viral RNA is not sufficient to demonstrate whether actual virus is present. This study [1] did not include assays to detect live virus, and so results and conclusions about virus positivity, virus loads, and infection rates should be interpreted as referring specifically to the presence or absence of viral RNA which may not accurately indicate the presence or absence of virus. The authors acknowledged that this was a shortcoming of the study but commented that detection of viral RNA has been used as an indicator of viral replication in other published studies, hence in their view it was a valid indicator of the antiviral effect of ivermectin.

There was an error in [1], in the sentence, “Adult mosquitoes aged between three and five days post emergence from larvae were used as experiment objects.” In this sentence, ‘larvae’ should be replaced with ‘pupae’.

## Supporting information

S1 File RT-qPCR data. Primary data obtained from the PCR machine (BIO-RAD CFX96) are on the sheets titled, “RT-PCR process Records #” and “Ct Value #”. The “RT-PCR process Records #” sheets record the process of reaction during the detection of each sample in the machine, and the “Ct Value #” sheets record the Ct Value for each sample, which is automatically calculated by the machine, based on the process of reaction. All the corresponding Ct values were copied to the sheet titled the summary file provided as S2 File with this notice. The Reaction holes on the “RT-PCR process Records” and “Ct Value” sheets are corresponding to each other. On the “Ct Value” sheets, the Cq standard deviation values were automatically calculated by the detection machine, each reports a calculation based on the Cq value for an individual well and hence all standard deviations are reported as zero. In column E (“Sample ID”) of the “Ct Value” sheets, samples are identified according to the following label convention: [concentration]-[subgroup]-[sample number]. In other words, a ‘2-1-19’ label in column E (File: Row Data 1) designates that data in that row represent the result for the 2ng/ml concentration, subgroup 1, sample 19. On the Data Analysis worksheet this is the 19th value in the Ct Value column for the 2 ng/ml group indicated as 2-1. The third sheet in each “Row data #” file includes information about the PCR run.
(ZIP)

S2 File Summary and analysis of RT-qPCR data. File with the summary data and analyses reported in the article. The summary data in this file was derived from the individual-level data in S1 File. There are several samples for which identical results are listed (Ct 40.01, log10 Value 1.3327482). The corresponding author explained that no DENV-2 was detected by RT-PCR for these samples. For the purpose of data analysis, negative samples were assigned Ct values of 40.01 according to the detection kit.
(XLSX)
